# Gasdermins: New Therapeutic Targets in Host Defense, Inflammatory Diseases, and Cancer

**DOI:** 10.3389/fimmu.2022.898298

**Published:** 2022-07-01

**Authors:** Laura Magnani, Mariasilvia Colantuoni, Alessandra Mortellaro

**Affiliations:** San Raffaele Telethon Institute for Gene Therapy (SR-Tiget), IRCCS San Raffaele Scientific Institute, Milan, Italy

**Keywords:** gasdermins, pyroptosis, inflammasome, host defense, cancer, therapeutics

## Abstract

Gasdermins (GSDMs) are a class of pore-forming proteins related to pyroptosis, a programmed cell death pathway that is induced by a range of inflammatory stimuli. Small-scale GSDM activation and pore formation allow the passive release of cytokines, such as IL-1β and IL-18, and alarmins, but, whenever numerous GSDM pores are assembled, osmotic lysis and cell death occur. Such GSDM-mediated pyroptosis promotes pathogen clearance and can help restore homeostasis, but recent studies have revealed that dysregulated pyroptosis is at the root of many inflammation-mediated disease conditions. Moreover, new homeostatic functions for gasdermins are beginning to be revealed. Here, we review the newly discovered mechanisms of GSDM activation and their prominent roles in host defense and human diseases associated with chronic inflammation. We also highlight the potential of targeting GSDMs as a new therapeutic approach to combat chronic inflammatory diseases and cancer and how we might overcome the current obstacles to realize this potential.

## Introduction

Gasdermins (GSDMs) are a group of proteins whose discovery has occurred gradually over the past two decades, beginning with the identification of *Gsdma1* in mice and soon followed by the mapping of the orthologous human gene *GSDMA* (1–3). To date, six human (*GSDMA-E* and *PEJVAKIN*) and ten mouse GSDM genes (*GsdmA1-3, GsdmC1-4, GsdmD, GsdmE*, and *PJVK*) have been identified, with distinct patterns of expression that are highly conserved between the two species (**Table 1**). Early clues on the functions of GSDMs came from this pattern of expression throughout the barrier epithelia of the esophagus, stomach, colon, bladder, vagina, and prostate, with some also being expressed in leukocyte subsets (GSDMA), lymphocytes (GSDMB), and myeloid cells (GSDMD) (3–5, 18).

**Table 1:** Comparison of gasdermin protein expression, function, associated diseases and therapeutics in mice and humans.

Gasdermin genes		Protein expression pattern		Function		Disease association	Therapeutics targeting	
Human	Mouse	Human	Mouse	Human	Mouse	Human	Drug	Indication
**GSDMA**	GsdmA1 GsdmA2 GsdmA3	Epithelial cells [bladder (2), gastrointestinal tract (4), skin (5)]	Epithelial cells [bladder (2), gastrointestinal tract (4), skin (5)]	Cell death	Cell death	Asthma (6) Systemic sclerosis (7)	–	–
**GSDMB**	None	Epithelial cells [gastrointestinal tract, bladder, airways, bone marrow, lymphoid tissues (4, 5)]	–	Pyroptosis	–	Asthma (8) Pro-tumoral activity (9)	–	–
**GSDMC**	GsdmC1 GsdmC2 GsdmC3 GsdmC4	Epithelial cells (4, 5) (skin, bladder, gastrointestinal tract, female tissues)	Epithelial cells (4, 5) (skin, bladder, gastrointestinal tract, female tissues)	Unknown	Unknown	No clear association	–	–
**GSDMD**	GsdmD	Leukocytes, respiratory system, gastrointestinal tract, bone marrow, liver, gallbladder (4, 5)	Leukocytes, respiratory system, gastrointestinal tract, bone marrow, liver, gallbladder (4, 5)	Pyroptosis	Pyroptosis	Hyper-activation in autoinflammatory diseases (CAPS, FMF) (10)	Dimethyl-fumarate (11) Disulfiram (12) α-NETA (13)	Hyper-inflammation Cancer
**GSDME**	GsdmE	Gastrointestinal tract, muscle tissues, genital tissues, endocrine tissues (4, 5)	Gastrointestinal tract, muscle tissues, genital tissues, endocrine tissues (4, 5)	Pyroptosis Anti-tumor activity	Pyroptosis Anti-tumor activity	Deafness (14) Tubulo- interstitial fibrosis (15) Cancer (16)	Disulfiram (12) Z-DEVD-FMK (15) miRNA combined with cetuximab (breast cancer) (17)	Hyper-inflammation Tubulo- interstitial fibrosis Cancer
**PJVK**	Pvjk	Transcript mainly expressed in male tissues and brain (4, 5)	Kidney, testis (4, 5)	Mitochondrial homeostasis Pexophagy (hypothesized)	Mitochondrial homeostasis Pexophagy (14)	Deafness (14)	–	–

The most important advance in our understanding of GSDM biology came in 2015 when two seminal articles simultaneously identified GSDMD as the primary executor of a type of pro-inflammatory programmed cell death known as pyroptosis (19, 20). Pyroptosis results from the formation of large numbers of GSDM pores that disrupt plasma membrane integrity, leading to cell death, and can be triggered by pathogen-derived or endogenous danger molecules (21). However, there is evidence of alternative outcomes of GSDM pore formation in which cells do not die, such as macrophage hyperactivation after nigericin stimulation or during *Salmonella typhimurium* infection in the presence of glycine (22), and a recent study illustrating a homeostatic role of GSDMD in mucus secretion in the gut (23). The factors that determine the extent of GSDM pore formation or its outcome are only just beginning to be understood. Nevertheless, it is clear that the balance between the two is central to the maintenance of health and in disease states.

Given the polarized outcomes of GSDM activation in cells, it is perhaps not surprising that there may be dire consequences when their function or regulation goes awry. While we now know that GSDMs are critical for protective responses to bacterial, fungal, and viral infections, recent research has also implicated these proteins in the pathogenesis of conditions as diverse as cancer (9, 24), alopecia (18), and asthma (6) (**Table 1**). This review highlights the most recent and important advances in the activation and innate immune functions of GSDMs and their roles in a range of pathologies. We will also examine the possibility of targeting this protein family therapeutically and reflect on the latest attempts to do so. Lastly, we will discuss the most pressing challenges and knowledge gaps that must be overcome to advance the field of GSDM research.

## Molecular Insights Into GSDM Activation

**Figure 1:**
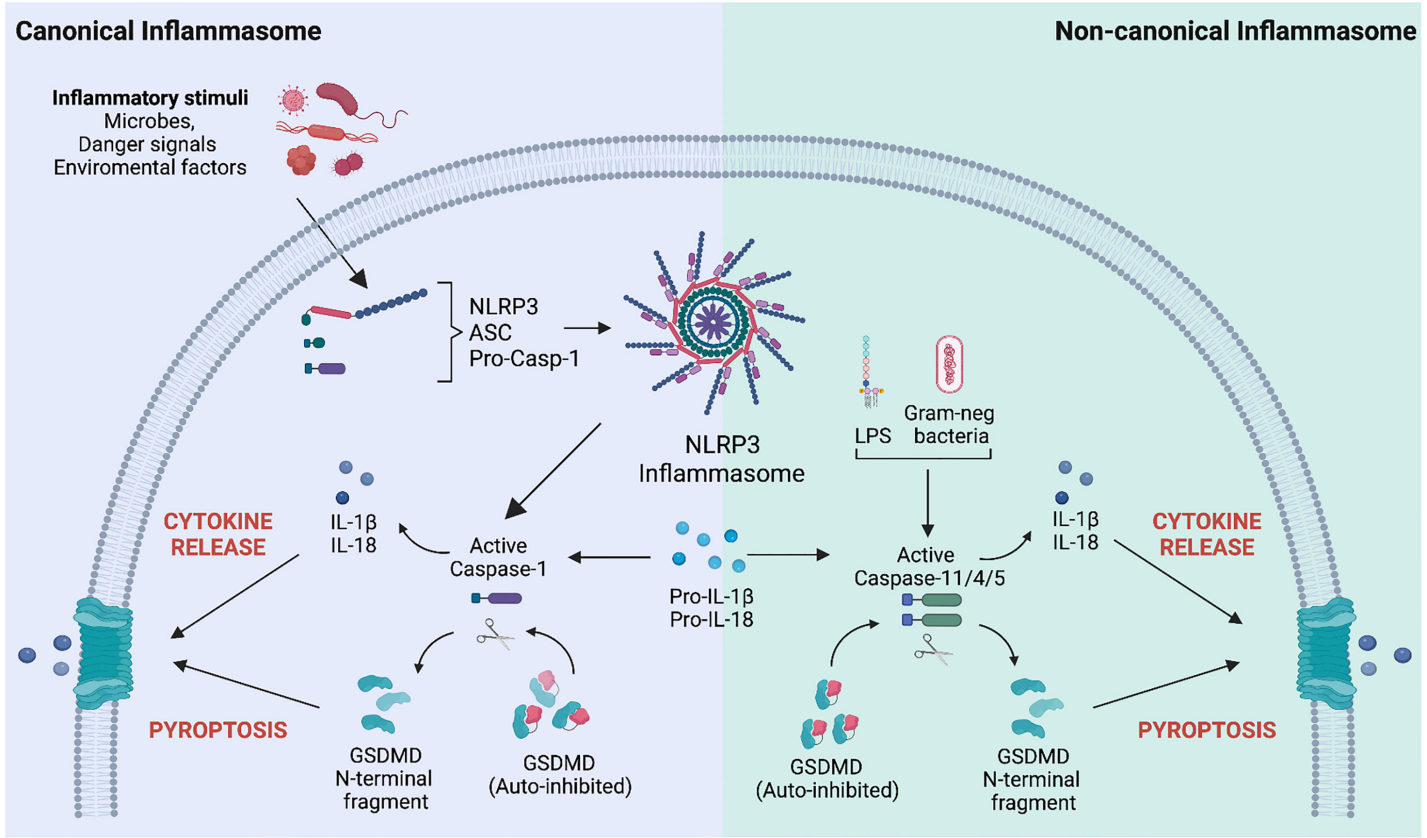
Mechanisms of Gasdermin D activation following canonical and non-canonical inflammasome stimulation. *Left.* Canonical inflammasome activation is a well-structured process involving several steps. Upon sensing specific inflammatory stimuli (microbes, danger signals, and environmental factors), the inflammasome sensor (*i.e.*, NOD-like receptor protein 3, NLRP3) recruits the precursor of caspase-1 (pro-caspase-1) *via* the protein adaptor apoptosis-associated speck-like protein containing a CARD (ASC). Upon inflammasome complex formation, pro-caspase-1 is activated by autocatalytic processing and converts the pro-IL-1β and pro-IL-18 precursors into their bioactive forms. Matured caspase-1 also cleaves the pore-forming protein gasdermin D (GSDMD), generating the N-terminal domain of GSDMD (GSDMD-NT), which relocates to the plasma membrane and oligomerizes to form a pore. Pore formation allows the release of mature IL-1β and IL-18 and drives a type of inflammatory cell death known as pyroptosis. *Right.* The non-canonical inflammasome pathway is activated by intracellular sensing of Gram-negative bacteria or lipopolysaccharides (LPS). It involves the activation of human caspase 4/5 or caspase-11 in mice. Active caspases directly process the pro-IL-1β and pro-IL-18 precursors and GSDMD. Like the canonical inflammasome activation mode, GSDMD pores allow the passive release of IL-1β/IL-18 cytokines and pyroptosis.

Following the discovery that GSDMD is central to pyroptosis, most studies on GSDM activation have focused on this family member and its role in myeloid cells, such as macrophages. Exposing macrophages to pathogen-associated molecular patterns (PAMPs), environmental particulates or toxins, or endogenous danger-associated molecular patterns (DAMPs) such as high mobility group box 1 (HMGB1), adenosine triphosphate (ATP), or cholesterol crystals, triggers the formation of a multiprotein complex, known as the inflammasome (**Figure 1**) (21). This type of canonical inflammasome activation leads to the activating autoproteolytic cleavage of pro-caspase-1, with two important consequences: caspase-1 processes the precursors of the pro-inflammatory cytokines IL-1β and IL-18 into their biologically active forms, and it also processes full-length GSDMD. This processing allows the N-terminal domain of GSDMD to mediate its oligomerization at the plasma membrane, thereby forming the pores that both allow IL-1β and IL-18 release while also – at least in some cases – leading to pyroptotic cell death (25, 26). Intracellular delivery of PAMPs, such as lipopolysaccharide (LPS), activates the non-canonical inflammasome, which also leads to IL-1β and IL-18 secretion and GSDMD processing, but, in this case, it is mediated by caspase-4 and -5 in humans, and caspase-11 in mice (**Figure 1**) (27). Nevertheless, there are exceptions to these rules. Inflammasome activation can selectively promote cytokine processing independently of pyroptosis as described in LPS-stimulated human monocytes (28) and *Salmonella*-infected mouse neutrophils (29) are resistant to pyroptosis while still being good producers of IL-1β. GSDMD pores serve as conduits for the passive release of IL-1β from hyperactivated but living macrophages in response to bacteria and DAMPs (30, 31). Cell vitality can be preserved by removing GSDMD pores *via* the transient recruitment of the endosomal sorting complexes required for transport (ESCRT) machinery at the plasma membrane (32). The ESCRT system is essential for many cellular processes, including membrane bending and fission reactions during the multivesicular body pathway, budding of enveloped viruses, membrane rupture phase in cytokinesis, and membrane repair. In particular, the ESCRT-III complex has been associated with all the ESCRT-mediated membrane patching functions (33). Ca^2+^ influx through GSDMD pores attracts the ESCRT-III complex at the damaged plasma membrane to remove GSDM pores and initiate the repair (32). Indeed, ESCRT-III protein inhibition enhanced IL-1β secretion and pyroptosis in human and mouse macrophages following inflammasome activation (32). These findings identify a fundamental role of the ESCRT complex in limiting GSDM-dependent pyroptosis cell death and cytokine release.

**Figure 2:**
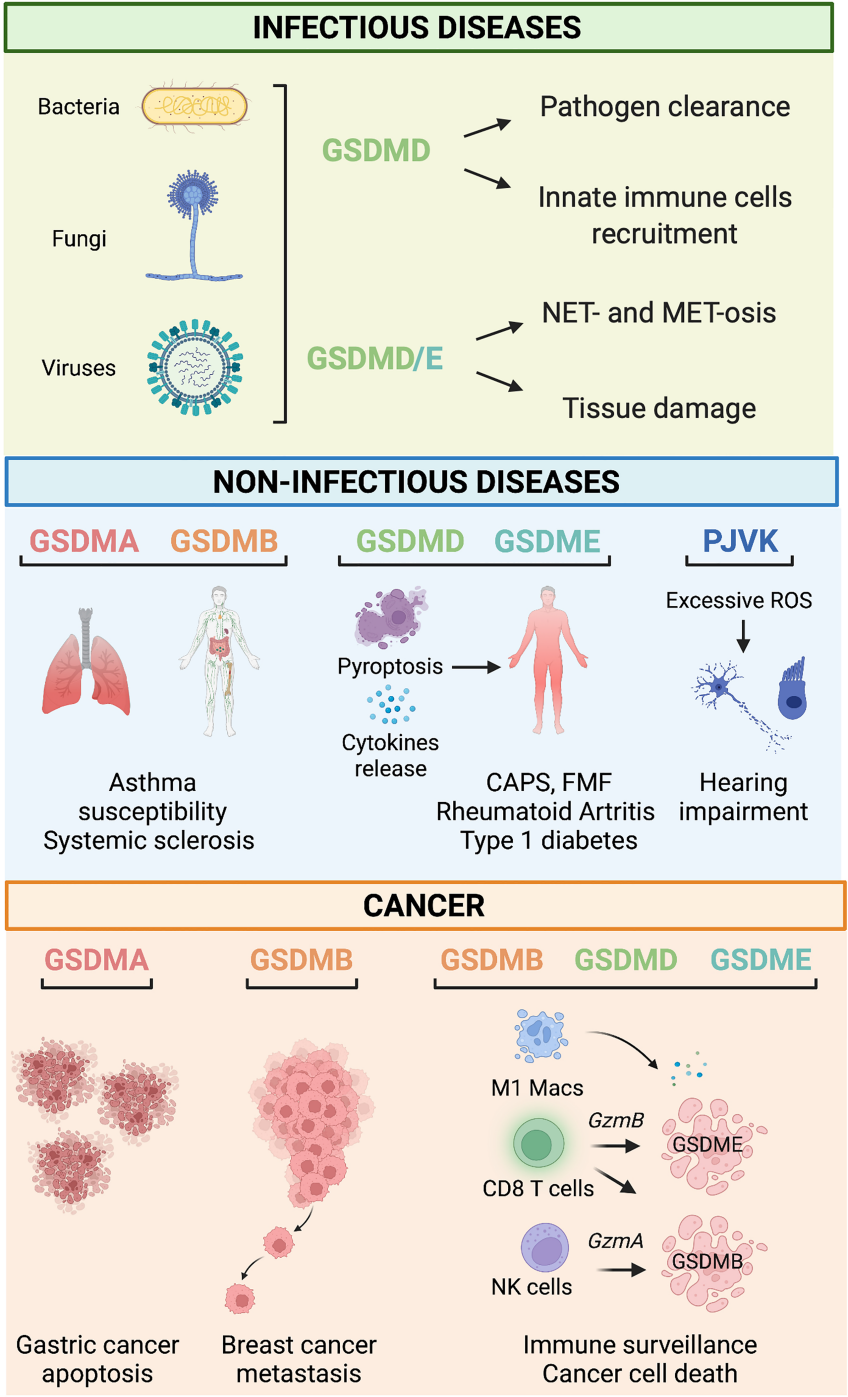
Gasdermin-related functions in infectious and non-infectious diseases and cancer. *Infectious diseases.* During infections, GSDM-mediated pyroptosis is, in general, beneficial for the host because it leads to successful pathogens eradication by mediating innate immune cell recruitment, NET- and MET-osis. However, under some circumstances, detrimental pyroptosis occurs which causes unwarranted and permanent tissue damage. *Non-infectious diseases.* GSDMA and GSDMB polymorphisms are associated with asthma susceptibility, bronchial hyperresponsiveness, and increased risk of multiple sclerosis. GSDMD and GSDME mediate pyroptosis and cytokine release associated with many immune-mediated inflammatory diseases such as Cryopyrin-associated periodic syndrome (CAPS), Familial Mediterranean fever (FMF), rheumatoid arthritis, and type 1 diabetes. *PJVK*-deficiency triggers excessive generation of reactive oxygen species (ROS), which cause hearing impairment due to cell death of auditory cells and sensory neurons. *Cancer.* GSDMA reduces the growth of gastric cancer cells by promoting apoptosis. On the other hand, GSDMB overexpression has been linked to metastatic and invasive properties of breast cancer cells. Tumor-infiltrating immune cells (*i.e.*, M1 macrophages, CD8^+^ T cells, and NK cells) promote cell death of cancer cells *via* a mechanism mediated by the granzyme-GSDMB/D/E pathway.

GSDM activation can also be achieved by proteases other than caspases under specific circumstances. For example, granzyme B, which is released in cytotoxic granules by CD8^+^ T lymphocytes and passed into target cells *via* perforin pores, can process and activate GSDME, leading to the target cell pyroptosis (16) (**Figure 2**). Similarly, granzyme A released by cytotoxic NK and CD8^+^ T cells into target cells can cause pyroptotic killing mediated by GSDMB (34). Furthermore, the neutrophil-specific serine protease elastase can produce a fully active GSDMD, resulting in NETosis, vital for effective pathogen clearance through neutrophil extracellular traps (NETs) formation (35, 36).

Alongside GSDM activation *via* the PAMP/DAMP-mediated inflammasome route or the alternative immune protease route, recent studies have uncovered links between the apoptotic program and GSDM-mediated pyroptosis. The extrinsic apoptotic pathway activates caspase-8, which can cleave GSDMD to elicit lytic cell death during infection of cells with *Yersinia spp* (37, 38). At the same time, other studies showed that GSDME is cleaved by the apoptotic caspase-3 in human cell lines *in vitro*, whose upstream activation can be triggered by etoposide or vesicular stomatitis virus (VSV) infection (39). The broader physiological/pathological relevance of these findings is yet to be fully explored, but together these studies illuminate multiple pathways whose activation can converge on GSDM activation and downstream pore formation. It will be a topic of much interest to understand which pathways predominate in different situations and whether they result in different outcomes for pore-bearing cells.

## Non-Pyroptotic Effector Functions of GSDMs

Some reports have also highlighted the non-pyroptotic functions of GSDMs in host defense against pathogens, which is an area of rapid development within the field. In bacterial infections, NET formation is a dominant mechanism of pathogen clearance, and Sollberger et al. recently revealed the role of GSDMD in promoting nuclear envelope permeabilization and extrusion of chromatin and other proteins to form NETs in both human and mouse neutrophils (36). A similar mechanism has now also been described in macrophages, where caspase-1-mediated GSDMD cleavage promotes membrane permeabilization, leading to “METosis,” a type of macrophage cell death characterized by the release of NET-like chromatin webs that capture extracellular bacteria (40). The significance of this new role for GSDMD is yet to be assessed more widely, but these findings are intriguing nonetheless.

Alongside assisting in NET- and MET-osis, work by Zhang et al. has uncovered a novel role for GSDMD in mucus secretion in the murine digestive tract during both homeostasis and pathogenic bacterial infection, which was required for effective clearance of enteric pathogens in mice (23). It is not yet clear how/whether this observation translates to humans. Still, this study provides the first indication of a homeostatic role for GSDMD within the intestinal epithelium and further evidence of the importance of GSDMs that extends beyond pyroptosis and into steady-state functions relating to pore-mediated secretory pathways that are yet incompletely defined.

## The Role of GSDMs in the Innate Immune Response to Infection

GSDMs are critical innate immune factors that promote inflammation to fight bacterial, fungal, and viral infections, which they achieve by executing pyroptosis and allowing the release of IL-1β and IL-18 to the extracellular space (**Figure 2**). Recent studies have hinted at further roles around the intersection of innate and adaptive immunity and at specialized GSDM pathways within neutrophils and macrophages. This section will summarize the known pyroptosis and non-pyroptosis-related functions of the GSDM family members during infections.

### Bacterial Infections

GSDM-mediated pyroptosis of infected cells releases numerous PAMPs and DAMPs, which actively promote phagocyte recruitment to the site of inflammation to mediate pathogen clearance. Human and mouse macrophages undergo pyroptosis upon infection with bacteria, as seen with *Yersinia* spp. (41), *Escherichia coli* (42), *Brucella* spp. (43), *Burkholderia thailandensis* (44), and *Salmonella enterica* spp. (45). During these infections, bacteria-derived molecules act as immunological “danger signals” and induce the inflammasome activation that drives GSDMs processing. Recent work identified *Staphylococcus aureus*-derived α*-*hemolysin (46) and lipoprotein 19 from *Brucella* bacteria as novel activators of the NLRP3 inflammasome and GSDMD processing. *Brucella* bacteria lipoprotein 16 leads to GSDME cleavage (43). However, in many cases, the precise pathogenic trigger is unknown.

Direct evidence of the importance of pyroptosis has been demonstrated recently by a study showing that non-canonical inflammasome activation through the caspase-11/GSDMD axis is critical for killing *Brucella abortus* inside the replicative niche (47). Indeed, *Gsdmd*- or *caspase-11*-deficient mice were more susceptible to *Brucella abortus* infection, probably due to defective recruitment of neutrophils, dendritic cells, and macrophages in spleens of infected mice (48). Other GSDM-mediated mechanisms have been shown to promote effective pathogen clearance by preventing bacterial replication. A cross-talk between GSDMD and autophagy has been proposed based on the observation that *Gsdmd*-deficient mice have an impaired autophagic process upon pulmonary infection with *Burkholderia cenopacia.* Indeed, less LC3-II conversion was observed in lung homogenates of infected *Gsdmd*-deficient mice (47). GSDMD-mediated direct killing of *Burkholderia thailandensis* was also demonstrated *in vitro* (44).

GSDMs can also alert tissue-resident cells surrounding the site of infection. *Yersinia* spp. infection triggered neutrophil and macrophage pyroptosis *via* a GSDMD/E-mediated pathway (38). Moreover, Chen et al. uncovered a crucial role of GSDME activation by the acetyltransferase YopJ of *Yersinia pseudotuberculosis* in the *in vivo* killing of infected neutrophils as a beneficial mechanism for the host (49). Moreover, neutrophils leverage NETs release to achieve successful pathogen clearance, and some evidence suggests that this occurs in a GSDM-dependent fashion (16, 33). For example, *Gsdmd*-deficient mice exhibited delayed neutrophil death and enhanced cytotoxic activity against *Escherichia coli* infection *in vivo*, further corroborating the GSDMD role for fine-tuning the immune response (33). As the involvement of GSDMs in METosis has only recently been described (49), it is not yet clear whether GSDM-driven over-activation of this process has relevance during infection, but this will be of great interest to study.

Pyroptosis is not always beneficial for the host. For example, the excessive inflammatory response caused by *Staphylococcus aureus*-induced pyroptosis is the leading cause of bovine mastitis, driving the damage of mammary tissue and epithelial cells (50). It has yet to be seen whether similar mechanisms operate in human mastitis, which is also predominantly caused by *Staphylococcus* spp. Craven et al. showed that α*-*hemolysin triggered the NLRP3 inflammasome in mouse and human monocytes, causing an enhanced IL-1β and LDH release (46). In human sepsis, Esquerdo et al. uncovered higher expression levels of inflammasome genes in patients who died of sepsis than those who survived (51), hinting at the possibility of GSDM involvement, but this has yet to be formally tested. However, in support of this notion, there is an earlier work showing that *Gsdmd*-deficient mice are protected from LPS-induced septic shock (20) and that administration of necrosulfonamide, which blocks GSDMD pore formation, lowers the level of systemic pro-inflammatory cytokines and prolongs survival in a murine model of lethal sepsis (52).

### Fungal Infections

Inflammasome activation also operates during opportunistic fungal infections caused by *Aspergillus fumigatus* (53) and *Candida albicans* (54). Although inflammasome involvement during fungal infections was already established (55, 56), the role of GSDMs is still incompletely clarified. GSDMD-mediated pyroptosis is one of the leading cell death mechanisms occurring upon fungal infections. Upon *Aspergillus fumigatus* or *Candida albicans* infection, mouse and human macrophages activate inflammasome and undergo PANoptosis (pyroptosis, apoptosis, and necroptosis) *via* a mechanism that requires the innate immune sensor Z-DNA binding protein 1 (ZBP1) as an apical sensor of fungal infection (57). Yan et al. revealed a key role of GSDMD in eradicating *Aspergillus fumigatus*, showing that Toll-like receptor 2 was required for the expression of GSDMD, IL-1α, and IL-1β, thereby modulating the pyroptosis (53).

The role of GSDMD-induced pyroptosis has been recently elucidated in a mouse model of *Candida albicans* keratitis, a sight-threatening corneal infection. Expression levels of NLRP3, caspase-1, IL-1β, and GSDMD, and pyroptotic cell death were significantly increased in *Candida albicans*-infected corneas (54). shRNA-mediated NLRP3 knockdown in corneas reduced inflammation and clinical scores, indicating that NLRP3 inflammasome activation is involved in the progression of *Candida albicans* keratitis (54). Further investigations are needed to elucidate the exact activation mechanisms and effector actions of GSDMs throughout fungal infections.

### Viral Infections

GSDMs are involved in the host response to viral infections, and there are examples showing the beneficial effects of GSDM-mediated pyroptosis in aborting viral replication in infected cells (58). *Gsdmd*-deficient mice were markedly more susceptible to rotavirus infection compared to wild-type, showing increased viral load and diarrhea (59). A recent paper highlighted that antibody-opsonized respiratory syndrome coronavirus 2 (SARS-CoV-2) virions triggered inflammation and pyroptosis in human blood monocytes of patients with COVID-19 *via* the NLRP3/AIM2 inflammasome pathway leading to caspase-1 and GSDMD processing. Lung macrophages also showed activated inflammasomes, suggesting that GSDMD-mediated inflammatory cell death restricts viral production but causes systemic inflammation that contributes to COVID-19 pathogenesis (60). In a lethal model of H5N1 influenza virus infection in non-human primates, histopathological sections of infected lungs showed significant pulmonary infiltration of neutrophils exhibiting upregulated expression of NETosis-related genes (61). Moreover, viral-activated macrophages and alveolar epithelial cells possibly contributed to the pathological outcomes through enhanced GSDMD-mediated pyroptosis and uncontrolled inflammasome activation (61).

The role of GSDMs in anti-viral cytokine release during viral infection is also beginning to be revealed. Type I interferons (IFNs) are well-characterized anti-viral agents, and new data showed that GSDMD mediates the non-canonical release of IFN-β and an enhanced response to IFN-stimulated genes during transmissible gastroenteritis virus and porcine delta coronavirus (pDCoV) infection *in vitro* (62). Alongside type I IFN, a recent study revealed a role for caspase-3-dependent cleavage of GSDME in the release of IL-1α from keratinocytes infected by VSV (63). This last study is the first to elucidate an anti-viral role for GSDME, calling for further investigation of this family member in the field of viral interactions.

Detrimental GSDMD activation was revealed in a STAT-1-deficient mouse model susceptible to norovirus (MNV) infection. MNV-infected macrophages and intestinal epithelial cells showed increased NLRP3 inflammasome activation, IL-1β maturation, and GSDMD-dependent pyroptosis, leading to gastrointestinal inflammation and increased lethality (64). Similarly, hepatitis C virus (HCV) caused hepatocyte pyroptosis soon after infection, but in the absence of GSDMD, HCV activated a caspase-3-mediated apoptotic program. On the other hand, caspase-3 knockout led to a reduced caspase-1 activation, suggesting that pyroptosis and apoptosis cooperate to enhance virus release from infected cells, thus contributing to liver pathogenesis (65). Concomitant apoptosis and pyroptosis also occurred in macrophages infected with the Zika virus, which may significantly impact viral pathogenesis in humans (66).

Viruses have evolved mechanisms to subvert the innate immune response, serving as the first line of defense. Accordingly, recent studies have uncovered multiple viral strategies to disable GSDM-mediated pore formation and pyroptosis. Influenza A virus avoids extensive pyroptosis by activating caspase-8-mediated processing of caspase-3, leading to the formation of the GSDMD p20 fragments, which are unable to form pores (67). In contrast, VSV infection activated GSDME in a caspase-3-dependent manner in keratinocytes. Mechanistically, VSV hijacked protein synthesis, which depletes host cells of anti-apoptotic proteins, such as BCL-2 family members, resulting in caspase-3-initiated GSDME-mediated pyroptosis (63). In addition, the viral protease pS273R of the African swine fever virus induced the processing of GSDMD into two pieces that cannot induce pyroptosis of the infected cells (68). The Nsp5 protease of porcine and human coronaviruses, including SARS-CoV-2, cleaved GSDMD into peptides unable to trigger pyroptotic cell death (69). Alternatively, SARS-CoV-2 nucleocapsid protein prevented GSDMD processing by binding to its linker region (70).

Together, these studies reveal a role for GSDMD-mediated pyroptosis in a wide range of infections and in several viral infections where immune evasion strategies are coming to the fore. This is a relatively new field of investigation. The examples listed here likely represent only a fraction of the actual number of infections in which pyroptosis is a key aspect of host defense. Further analysis of pathogen-mediated anti-GSDM/anti-pyroptosis strategies may reveal novel ways of interfering with this pathway therapeutically.

## Dysregulated GSDM Activation in Non-Infectious Diseases

Given their critical and finely balanced roles in maintaining homeostasis and fighting infection, it is perhaps not surprising that aberrant GSDM activation can contribute to disease pathways (**Figure 2**). We have known for some time that single nucleotide polymorphisms in *GSDMA* and *GSMDB* are associated with asthma susceptibility, elevated IgE levels, and bronchial hyperresponsiveness in children with asthma; and that mice transgenic for the higher risk human *GSDMB* allele develop similar airway-hyperresponsiveness (8). More recently, a meta-analysis of genome-wide association studies in humans identified *GSDMA* and *GSDMB* variants associated with systemic sclerosis (7): macrophages from patients with systemic sclerosis who carried the risk variant rs3894194 showed GSDMA over-expression (71), but clear links between the functions of these less well-studied GSDMs and the pathophysiology of the disease remain to be elucidated.

As our understanding of the significance of GSDMs in inflammatory diseases grows, many studies are re-examining NLRP3-inflammasome-mediated conditions and asking whether GSDMs are involved. Cryopyrin-associated periodic syndromes (CAPS) result from gain-of-function mutations in the NLRP3 gene (10), and mouse models of CAPS exhibit excessive activation of GSDMD, which significantly contributes to the disease etiology (72). Another study showed that *Gsdmd* ablation in a mouse CAPS model rescued lethality, skin, spleen abnormalities, leukocytosis, and anemia (73). Similar results were seen in a mouse model of Familiar Mediterranean fever (FMF). The inflammasome-driven auto-inflammation that characterizes the condition was entirely ameliorated by the deletion of *Gsdmd* (74). However, there is some evidence from mice that, in the absence of GSDMD, GSDME can functionally compensate, maintaining high levels of IL-1β and IL-18 during constitutive NLRP3 inflammasome stimulation (12). The relevance of the GSDME pathways in conditions where inflammasome hyperactivation is implicated should be a priority research area.

Several GSDM family members have also been associated with other diseases characterized by immune dysregulation, including inflammatory bowel disease (IBD). GSDMA and *GSDMB* are susceptibility genes (75, 76) but with yet-undefined roles in the condition. However, in mice, there is evidence that GSDMD is required for microbiota-stimulated IL-18 release in the inflamed gut (77) and that it also mediates the non-lytic release of intestinal-epithelial-cell-derived inflammatory IL-1β-containing vesicles (78). In another auto-inflammatory disease, rheumatoid arthritis, there is a positive correlation between GSDME expression and TNF-induced pyroptosis mediated by the caspase-3/GSDME pathway in patients’ monocytes. *Gsdme* deficiency decreases disease incidence and severity in a collagen-induced arthritis mouse model (79). Interestingly, the same caspase3-dependent GSDME-mediated pyroptotic pathway is seemingly involved in type 1 diabetes progression and diabetic nephropathy, a severe co-morbidity of diabetes (15). Future studies should reveal whether this route towards pyroptosis is more broadly activated in such conditions.

The GSDM family member PJVK is expressed in the sensory cells and neurons of the auditory part of the inner ear (4). Indeed, PJVK is a peroxisome-associated protein required for oxidative-stress-induced proliferation in sensory hair cells (80). Loss-of-function mutations in human and mouse *PJVK* are associated with hearing defects caused by impaired peroxisome degradation induced by excessive noise (14). Noise overexposure in *Pjvk*-deficient mice causes marked reactive oxygen species production, diminished antioxidant response, and inefficient autophagic degradation of peroxisomes (14). Gain-of-function *GSDME* variants are also related to hearing impairment by enhancing a detrimental cytotoxic activity of GSDME (81).

These studies reveal multiple roles of different GSDM family members in various auto-inflammatory and auto-immune conditions. However, in many cases, the picture is incomplete. Our ability to delve further into disease mechanisms is limited by our lack of knowledge of the upstream and downstream events of the less well-studied members of the GSDM family.

## The Role of GSDMs in Cancer

The ability of GSDMs to activate inflammatory pathways, leading to the recruitment of both innate and adaptive immune effector cells, and to execute cell death, has led to an increasing interest in their potential role in cancer (**Figure 2**). Multiple studies have reported the over/under-expression of GSDM proteins in various cancers. Still, a clear pattern has yet to emerge, and, again, a lack of basic knowledge limits our ability to interpret some of the data. For example, *GSDMA* is expressed at a lower level in human esophageal and gastric cancers than in normal tissue (82). Although this observation unveils a possible tumor suppressor role for GSDMA in gastric cancer, its biological significance is yet to be revealed. Epigenetic mechanisms such as methylation processes were at the basis of *GSDMA* silencing in the mucus-secreting pit cells of the gastric epithelium (83). Moreover, *GSDMA* expression was regulated by the transcription factor LIM domain only 1 induced by transforming growth factor-β (83, 84). On the other hand, GSDMB is overexpressed in gastric, head, and neck cancers (82). In the case of breast cancer, we understand a little more of the possible role of GSDMB: high GSDMB expression in tumor cells from patients with ERBB2-positive breast cancer correlates with poor prognosis, increased risk of metastasis and decreased survival (85), which may be linked to earlier *in vitro* observations of increased invasiveness and metastatic ability of GSDMB overexpressing immortalized MCF7 tumor cells (9). Hergueta-Redondo and colleagues suggested that the increased migration and invasion capacity of GSDMB-overexpressing cells could be associated with activation of Ras-related C3 botulinum toxin substrate 1 (Rac-1) and cell division cycle 42 (Cdc-42) GTPases (9).

A recent pan-cancer analysis more comprehensively examined patterns of GSDM expression and assessed their relationship with key immune parameters in tumors: the most striking finding marked GSDMD overexpression in many cancer types, including gastric, colon adenocarcinoma, and breast cancers, which – in contrast to the negative correlations with GSDMB in breast cancer – was associated with increased numbers of potentially beneficial tumor-infiltrating CD8^+^ T cells, M1 macrophages, and NK cells (82). In endometrial cancer, GSDMD expression is significantly higher in cancer cells than in healthy endometrial tissue, and its expression is linked with anti-tumor immune properties and a more favorable prognosis (86). Similarly, GSDME expression in patient-derived breast cancer cells correlates with increased survival of patients because of pyroptosis triggered by granzyme B released by cytotoxic T cells; accordingly, its absence is associated with reduced lifespan and a high risk of metastasis (16). There is emerging evidence that sub-optimal expression of GSDME, at least in some cancers, is due to hypermethylation of its promotor: Ibrahim et al. identified key sites of *GSDME* hypermethylation in colorectal cancer cells but not normal colorectal tissue (87), drawing fresh attention to an early study that showed demethylation by 5-aza-2’-deoxycytidine restored GSDME induction in a breast cancer cell line (88).

Together, these observations support the notion that GSDMD and GSDME act as tumor suppressors through programmed cell death and immune cell recruitment and propose them as prognostic markers for tumor progression and patient survival. It also seems likely that at least GSDMB plays a role in cancer progression. In this case, it appears to be detrimental, perhaps even acting against other GSDM family members within the same types of cancer (**Figure 2**). Further studies looking at all GSDM family members together will be required to understand better the complex interactions between tumor cells, pro- and anti-cancer GSDMs, the immune microenvironment, and their effect on patient outcomes.

## Therapeutic Targeting of GSDMs

It is now clear that dysregulated activation or expression of GSDMs can either drive or contribute to the pathology of unrestrained inflammation during infection, autoinflammation/autoimmunity, and cancer. This realization has led to a surge in interest in the possibility of targeting GSDM family members for therapeutic benefit and to some unexpected insights into the effects of already licensed drugs.

In the case of inflammatory disorders, the multiple sclerosis drug dimethyl-fumarate promotes anti-inflammatory immune responses, but until recently, much of its mechanism of action has been unclear. Studies in mice have now revealed that the drug blocks caspase-mediated GSDMD activation, thereby suppressing pyroptosis *in vitro* and ameliorating symptoms in murine models of multiple sclerosis and FMF (11). The same drug is undergoing trials to treat ischemic stroke, (89). An FDA-approved medication for alcoholism, tetraethyl thiuram disulfide (disulfiram), which produces an aversive physical response to alcohol ingestion by preventing its oxidation, has also recently been found to inhibit GSDMD activation. When administered to mouse CAPS models, this drug blocked IL-1β maturation and pyroptosis and prevented the autoinflammatory spectrum disorder associated with inflammasome hyperactivation in these mice (90). Possibilities for targeting the caspase-3/GSDME axis are on the horizon, with a recent pre-clinical study reporting the successful use of Z-DEVD-FMK to ameliorate tubulointerstitial fibrosis and declining renal function in diabetic mice (15). In summary, the idea of targeting GSDMs for the treatment of inflammatory disorders is in its infancy, but these studies represent a promising indication of future potential.

Pre-clinical GSDM-targeted therapies for cancer are also an emerging area of research. While apoptotic pathways are often disabled in cancer cells, the caspase-8-caspase-3-GSDME axis leading to pyroptosis may be operative (91): aiming to exploit this, Lu et al. showed that intratumoral delivery of adeno-associated virus expressing the N-terminal domain of GSDME induced pyroptosis in glioblastoma and breast cancer cells, leading to tumor regression and prolonged survival in preclinical cancer models (92), but this has yet to be tested in humans. Some conventional chemotherapeutic drugs, such as cisplatin and doxorubicin, also cause pyroptosis in cancer cell lines by activating the caspase-3-GSDME pathway (12, 93); while micro-RNA-mediated reactivation of GSMDE expression, in combination with cetuximab, effectively induced pyroptosis of tumor cells and reduced tumor volume in a mouse model of aggressive triple-negative breast cancer (17). GSDMD has also been investigated as a potential therapeutic target in cancer: treating endometrial or ovarian cancer cells with α-NETA, a reversible choline acetylcholine transferase inhibitor, induced pyroptosis *via* the GSDMD/caspase-4 pathway, and reduced tumor growth in mice (13). In addition, paclitaxel, a common chemotherapeutic drug, combined with ruthenium (II) polypyridyl complex, an anti-tumoral compound, led to extensive GSDMD-mediated pyroptotic cell death in a taxol-resistant cervical cancer cell line.

Whilst all the studies to date have focused on the pre-clinical properties of GSDM-targeting, the data are encouraging, and several clinical studies are planned for the coming years. In support of these studies, more basic data are needed to understand the roles of the different GSDM family members in cancer and how they interact in specific situations.

## Current Challenges and Future Directions

The discovery that GSDM family proteins regulate pyroptosis is one of the significant breakthroughs in the innate immune cell biology of the past decade. The concept that canonical and non-canonical inflammasome activation by microbial and endogenous signals regulates IL-1β/IL-18 maturation and release and the death of innate immune cells has been discussed for a long time: but the basis of the underlying mechanism was unknown. Through a series of careful molecular studies, we now know that GSDM proteins are the main executioners of pyroptotic cell death *via* a process that requires their proteolytic cleavage by inflammatory caspases. Since these caspases are expressed in various cell types, including epithelial cells and keratinocytes, it is evident that GSDM-mediated pyroptosis also extends to non-immune cells. Increasing evidence shows that GSDMs are also involved in various physiological processes, including passive secretion of proteins and molecules that act as alarmins for the immune system, ROS production, MET/NETosis, and mucus layer formation in the gut. In recent years, a clear role of GSDM has emerged in numerous diseases; however, the exact mechanisms of how these proteins influence disease pathogenesis remain to be elucidated.

Among the questions that remain to be answered, some appear more pressing. For example, do the GSDMD features of auto-inhibition, activation, and pore formation also apply to the other members of the GSDM family? At present, the mechanisms of activation and the biological functions of GSDMA, GSDMB, and GSDMC are mainly unknown. Furthermore, it is paramount to uncover other upstream regulators of GSDM activation in immune and non-immune cells and to fully characterize the range of downstream consequences. The idea that GSDM pore formation is a terminal event leading to cell death is outdated, as GSDMD pores can also be exocytosed in the vesicles (32), and pore formation appears to be part of the normal mucin-granule release in the intestine (23). There may yet be other mechanisms of membrane recovery that we do not yet understand.

Several studies revealed the role of GSDMD in host defense against infections. GSDMs have also been associated with immune-mediated diseases, particularly those triggered by chronic inflammation. Mouse and human studies have pointed towards a role of GSDME in cancer tumorigenesis to such an extent that GSDM expression has been proposed as a biomarker for prognosis and response to treatment. Progress in the field has been rapid but – without exception – pre-clinical. Future studies must broaden our understanding of the triggers and functions of GSDM activation across homeostatic, pathogen-infected, and neoplastic tissues. Only then we will be able to manipulate GSDMs and realize the potential of therapeutics targeting their inflammatory or pyroptotic effects to treat human disease.

## Author Contributions

AM conceived the study and wrote the manuscript; LM collected the related manuscripts, wrote the manuscript, prepared the table, and revised the figures; MC prepared the figures and revised the manuscript. All authors approved the final manuscript.

## Funding

This work was funded by a grant from Fondazione Telethon (TIGET22-AM Core Grant) and the Else Kröner Fresenius Prize for Medical Research 2020.

## Conflict of Interest

The authors declare that the research was conducted in the absence of any commercial or financial relationships that could be construed as a potential conflict of interest.

## Publisher’s Note

All claims expressed in this article are solely those of the authors and do not necessarily represent those of their affiliated organizations, or those of the publisher, the editors and the reviewers. Any product that may be evaluated in this article, or claim that may be made by its manufacturer, is not guaranteed or endorsed by the publisher.
